# Reactive Oxygen Species-Responsive Chitosan–Bilirubin Nanoparticles Loaded with Statin for Treatment of Cerebral Ischemia

**DOI:** 10.34133/bmr.0097

**Published:** 2024-10-24

**Authors:** Raveena Nagareddy, Ja-Hae Kim, Ji-Hye Kim, Reju George Thomas, Kang-Ho Choi, Yong-Yeon Jeong

**Affiliations:** ^1^ Department of Biomedical Sciences, Chonnam National University Medical School and Hwasun Hospital, Hwasun, South Korea.; ^2^ Department of Nuclear Medicine, Chonnam National University Medical School and Hospital, Gwangju, South Korea.; ^3^ Department of Neurology, Chonnam National University Medical School and Hospital, Gwangju, South Korea.; ^4^ Department of Radiology, Chonnam National University Medical School and Hwasun Hospital, Hwasun, South Korea.

## Abstract

Cerebral ischemia impairs blood circulation, leading to elevated reactive oxygen species (ROS) production. A ROS-responsive delivery of drugs can enhance the therapeutic efficacy and minimize the side effects. There is insufficient evidence on the impact of ROS-responsive nanoparticles on ischemic stroke. We developed ROS-responsive chitosan–bilirubin (ChiBil) nanoparticles to target acute ischemic lesions and investigated the effect of atorvastatin-loaded ROS-responsive ChiBil. We randomly assigned rats with transient middle cerebral artery occlusion (MCAO) to 4 groups: saline, Statin, ChiBil, and ChiBil-Statin. These groups were treated daily via the tail vein for 7 d. Behavioral assessment, magnetic resonance (MR) imaging, evaluation of neuroinflammation, blood–brain barrier (BBB) integrity, apoptosis, and neurogenesis after stroke were conducted. In vitro, results showed nanoparticle uptake and reduced intracellular ROS, lipid peroxidation, and inflammatory cytokines (IL-6 and TNF-α). In vivo, results showed improved motor deficits and decreased infarct volumes on MR images in the ChiBil-Statin group compared with the Control group on day 7 (*P* < 0.05). Furthermore, the expression of inflammatory cytokines such as IL-1β and IL-6 was reduced in the ChiBil-Statin group compared with the Control group (*P* < 0.05). Improvements in BBB integrity, apoptosis, and neurogenesis were observed in the ChiBil-Statin group. The findings demonstrated that intravenous ROS-responsive multifunctional ChiBil-Statin could effectively deliver drugs to the ischemic brain, exerting marked synergistic pleiotropic neuroprotective effects. Therefore, ChiBil-Statin holds promise as a targeted therapy for ischemic vascular diseases characterized by increased ROS production, leading to new avenues for future research and potential clinical applications.

## Introduction

Large hemispheric infarctions caused by large vessel occlusion (LVO) constitute a critical cause of severe morbidity and mortality. Mechanical thrombectomy (MT) is currently the standard of care for acute stroke patients with LVO [1]. MT, which results in the complete recanalization of LVO, has become more prevalent over time [2]. However, despite successful recanalization rates of over 80% with MT, the rate of poor outcomes with functional dependence is around 50% among patients treated with MT [2]. Therefore, a better understanding of the essential factors contributing to cerebral ischemic damage would improve functional outcomes in cerebral ischemia–reperfusion and facilitate the clinical management and prognosis of stroke [3].

A considerable body of evidence suggests that oxidative stress is a key mechanism causing brain damage in cerebral ischemia–reperfusion [4]. The brain is highly susceptible to reactive oxygen species (ROS), which can induce damage due to low levels of protective antioxidants, high concentrations of peroxidizable lipids, high oxygen consumption, and high levels of iron that act as pro-oxidants under pathological conditions [5]. ROS produced due to ischemia/reperfusion can increase neuronal and blood–brain barrier (BBB) damage, leading to a lack of nutrient supply and causing a cascade of reactions such as a reduction in antioxidants, an increase in lipid peroxidation, and neuronal cell death. [6]. ROS have been implicated as one of the earliest and most important components of tissue injury in cerebral ischemia–reperfusion [7].

Therefore, in cerebral ischemia–reperfusion characterized by increased ROS production, nanoparticles designed to respond to ROS could be particularly effective [8]. ROS-responsive drug delivery systems can enhance therapeutic efficacy for the targeted brain region while minimizing systemic side effects [9]. Previously, we synthesized ROS-responsive chitosan–bilirubin (ChiBil) nanoparticles, which could be used for anti-inflammatory and ROS-responsive drug release [10]. Chitosan nanoparticles are known for crossing the BBB [11] and enhancing neuroprotective effects [12]; thus, they have been used for the treatment of cerebral ischemia [13]. Under cerebral ischemia conditions, bilirubin can act as a powerful antioxidant with anti-inflammatory effects [14] and a stimulus for drug delivery [15]. Water-insoluble bilirubin can be incorporated into nanoparticles, and a shift in solubility caused by ROS can disrupt the nanostructure, convert the bilirubin to the more water-soluble biliverdin, and lead to the release of the encapsulated drug [16].

Statins have been found to be beneficial for neuroprotection in clinical and animal studies [17–24]. Atorvastatin can exert neuroprotective effects through anti-inflammatory and anti-oxidative activity in acute cerebral ischemia–reperfusion injury. Previously, we demonstrated the therapeutic and neuroprotective effects of atorvastatin-loaded nanoparticles for treating cerebral ischemia–reperfusion [18]. In this study, we developed ROS-responsive ChiBil nanoparticles loaded with atorvastatin (ChiBil-Statin) for targeted delivery to the ischemic brain, which could exert synergistic effects through a combination of neuroprotective agents. We investigated the effect of ChiBil-Statin on rats with cerebral ischemia–reperfusion.

## Materials and Methods

### Materials

Glycol chitosan (molecular weight, ~82 kDa) was purchased from Chem Cruz (Dallas, TX, USA). Bilirubin was purchased from Cayman Chemicals (Ann Arbor, MI, USA). Methanol, 1-ethyl-3-(3-dimethylaminopropyl) carbodiimide (EDC), *N*-hydroxysuccinimide (NHS), IR780 iodide, and atorvastatin calcium were purchased from Sigma-Aldrich (St. Louis, MO, USA). Dimethyl sulfoxide (DMSO) was purchased from JUNSEI Chemical (Tokyo, Japan). Fetal bovine serum (FBS), Dulbecco’s modified Eagle’s medium (DMEM), and penicillin–streptomycin were purchased from Welgene Inc. (Daegu, South Korea).

### Synthesis and characterization of ROS-responsive ChiBil nanoparticles

#### Synthesis of ROS-responsive ChiBil nanoparticles

**Fig. 1.**
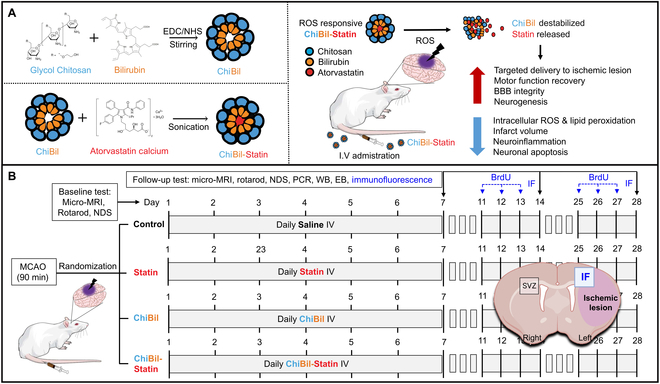
Nanoparticle synthesis and experimental scheme. (A) Micellar structure of ChiBil, loading of atorvastatin calcium, and its properties and effects in treating cerebral ischemia. (B) Experimental scheme. Rats with transient MCAO were randomly assigned to 4 groups: Control, Statin, ChiBil, and ChiBil-Statin. Each group received daily administration of the respective formulations intravenously for 7 d. Treatment effects were assessed using micro-MRI, rotarod test, NDS, PCR, Western blot (WB), Evans blue (EB), and immunofluorescence (IF) to evaluate functional recovery, BBB integrity, neuro-inflammation, apoptosis, and neurogenesis after stroke.

ROS-sensitive ChiBil nanoparticles were prepared following a protocol previously described (Fig. 1A) [10]. In brief, 33.33 mg of glycol chitosan was dissolved in 6 ml of distilled water, and 0.59 mg of bilirubin was dissolved in 1 ml of DMSO. The bilirubin solution was uniformly mixed with the dissolved glycol chitosan, followed by the addition of EDC and NHS for the binding of the COOH of bilirubin and NH_2_ of glycol chitosan. The entire mixture was stirred for 24 h and was dialyzed using a 3.5-kDa dialysis membrane (Spectra/Por, Spectrum Labs Inc., Phoenix, AZ, USA) against distilled water for 48 h to remove impurities and solvents. The solution was then sonicated for 10 min at a 10:5 pulse rate to form the micellar structure. The resulting solution was freeze dried to obtain ChiBil.

#### Loading of statin/IR780 in ChiBil nanoparticles

Atorvastatin loading into ChiBil micelles was performed using solvent evaporation [25]. We used methanol as an organic phase to load atorvastatin inside ChiBil. In brief, 3 mg of atorvastatin calcium was dissolved in 200 μl of methanol and uniformly mixed with 10 mg of ChiBil dissolved in 10 ml of distilled water by stirring for 12 h. The unloaded drug was removed by dialysis against water for another 12 h, followed by freeze drying. Subsequently, the oil-in-water emulsion method was used to load IR780 iodide dye into ChiBil, where 2 mg of dye was loaded into 10 mg of ChiBil.

#### Characterization of ChiBil-Statin nanoparticles

The hydrodynamic size and polydispersity index of ChiBil-Statin were measured using Zetasizer (Malvern Instruments, Malvern, UK). The instrument was used to measure the surface charge of the particles. Morphological analysis was performed by transmission electron microscopy (TEM). ChiBil nanoparticles were loaded with SPIONs (negative staining) and coated on 200-mesh carbon copper grids, and TEM was performed using JEM-2010 (JEOL, Tokyo, Japan). The maximum drug loading capacity was calculated using the same quantity of 10 mg of micelles. The encapsulation efficiency of atorvastatin calcium was assessed by breaking down the micelles in the presence of methanol and measuring the absorbance by ultraviolet (UV)–visible spectroscopy, which was calculated with the following equation:

$$\text{Encapsulation Efficiency}\%=\frac{\text{Amount of drug in nanoparticle}}{\text{Amount of drug feeded}}*100$$

#### ROS-responsive drug release study

ChiBil-Statin was dissolved in phosphate-buffered saline (PBS) at a concentration of 1 mg/ml, filled in a dialysis bag, and incubated in PBS or 100 mM 2,2′-azobis(2-amidinopropane) dihydrochloride (AAPH) at 37 °C in a shaking incubator at 80 rpm. The samples were taken in a timely manner from the PBS solution in which the samples were eluted. The same volume of fresh PBS was added to the removed portion. The samples were then diluted in methanol, and the statin concentration was determined by UV–visible spectrometry (Shimadzu, Kyoto, Japan) at 241 nm.

### In vitro experiments

All in vitro experiments were performed with the mouse BV-2 microglial cell line (American Type Culture Collection, Manassas, VA, USA). The cells were maintained at 37 °C in a 5% CO_2_ atmosphere. The cells were grown in DMEM-high glucose supplemented with 10% FBS and 5% penicillin–streptomycin as the culture medium.

#### Cell viability assay

Cells were seeded in a 96-well plate at 1 × 10^4^ cells per well. Once the cells were attached to the cell culture dish, the nanoparticles were added. After 24 h, MTS [3-(4,5-dimethylthiazol-2-yl)-5-(3-carboxymethoxyphenyl)-2-(4-sulfophenyl)-2H-tetrazolium; Promega, Madison, WI, USA] assay was performed to assess cell viability. The absorbance was measured using a multi-plate reader (TECAN, Mannedorf, Switzerland) at 492 nm.

#### Oxygen–glucose deprivation model

An oxygen–glucose deprivation (OGD) model for cerebral ischemia was induced in a Galaxy 48R hypoxia chamber (Eppendorf, Hamburg, Germany) with O_2_ levels at 1% and CO_2_ levels at 5%. The cells were treated with glucose-deficient DMEM supplemented with 10% FBS and 5% penicillin–streptomycin for 4 h and reperfused with normal medium under normoxia for 24 h.

#### Cell uptake study

Cells were treated with IR780-loaded ChiBil nanoparticles with and without OGD for 4 h. The cells were fixed with 4% paraformaldehyde, mounted with DAPI Gold Antifade Reagent (Invitrogen, Waltham, MA, USA), and imaged with a fluorescence microscope (Evos FL Auto 2; Life Technologies, Carlsbad, CA, USA).

#### ROS and malondialdehyde detection assay

Dichlorofluorescin diacetate (DCF-DA; Abcam, Cambridge, MA, USA) assay was performed to assess ROS reduction in OGD-treated cells using nanoparticles loaded with the drug, which was performed following the recommended protocol [26]. Images were taken using a fluorescence microscope (Evos M5000; Invitrogen, Waltham, MA, USA). Levels of malondialdehyde (MDA) (Elabscience, Houston, TX, USA) were assessed using a commercially available kit.

#### ELISA for inflammatory markers

Cells were cultured under both OGD and normal conditions, and supernatant samples were collected. Enzyme-linked immunosorbent assay (ELISA) kits for tumor necrosis factor-α (TNF-α) and interleukin-6 (IL-6) were purchased from Invitrogen (Waltham, MA, USA). The procedure was performed according to the protocol provided.

### In vivo experiments

All animal studies were performed using male Sprague–Dawley rats (8 weeks, 253 to 288 g) obtained from Samtaco Bio (Seoul, South Korea). A middle cerebral artery occlusion (MCAO) animal model was established by transient blocking of the left middle cerebral artery of the rats for 90 min with an intraluminal filament, followed by reperfusion for another 90 min. All rats were anesthetized with isoflurane (Forane Solution, Abbott, Green Oaks, IL, USA) in an O/NO mixture at a ratio of 30:70 using a gas mask. The procedure was performed with a thermostatically controlled heating pad to maintain the body temperature of the rats at 36.6 ± 0.5 °C. Only rats with a pretreatment neurological deficit score (NDS) of 2 or 3 were included in the study. According to the randomized group, saline, statin (3 mg/kg), ChiBil (weight equal to ChiBil-Statin), and ChiBil-Statin (3 mg/kg statin) were administered daily to the rats with transient MCAO through the tail vein for 7 d (Fig. 1B). Animal protocols were conducted following the Chonnam National University guidelines for the care and use of laboratory animals and were approved by the Institutional Animal Care and Use Committee (approval number: CNUHIACUC-22033). All laboratory experiments described in this article complied with the US Public Health Service Policy on the Humane Care and Use of Laboratory Animals.

#### In vivo magnetic resonance imaging

T2-weighted magnetic resonance imaging (MRI) was performed using the M7 Compact MRI System (Aspect Imaging, Shoham, Israel). The animals were anesthetized using isoflurane. The T2-weighted spin-echo MRI parameters were as follows: repetition time/echo time: 4,000/100 ms, inversion time: 100 ms, echo train length: 12, flip angle: 150°, number of slices: 20, slice thickness: 1.5 mm, field of view: 4 cm, no interslice gap, and matrix: 256 × 210. MRI was repeatedly performed on days 1, 3, 5, and 7 with the same rats (*n* = 8). Infarct volumes on the MR images were quantified using the imaging and analysis software MIPAV (version 11.1.0; NIH, Bethesda, MD, USA).

#### Behavioral study

We conducted behavioral tests at predetermined intervals to evaluate neurological recovery after treatment following MCAO. Experimental cohorts were categorized based on the NDS considering parameters such as level of consciousness (0 for normal, 1 for restless, 2 for passive, 3 for stupors), gait abnormalities (0 for normal, 1 for paw adduction, 2 for unbalanced walking, 3 for circling, 4 for inability to stand, 5 for lack of movement), limb tone (0 for normal, 1 for spastic, 2 for flaccid), and pain reflex (0 for normal, 2 for hypoactive, 3 for absent). A higher NDS indicated a more severe neurological condition [27].

Motor function impairment was assessed by the accelerated rotarod test. Each experimental group underwent the test at both baseline and 7 d after treatment. The rotating drum was gradually accelerated over an exercise period of 5 min, and the duration (in seconds) until the animal fell off was recorded. Each session comprised 5 consecutive trials, with a maximum time limit of 300 s. The mean latency to fall was calculated from the 5 trials.

#### Quantitative reverse transcription polymerase chain reaction

Total RNA was collected from the brain on day 7 using TRIzol (Invitrogen, Waltham, MA, USA) according to the manufacturer’s protocol. Quantitative reverse transcription polymerase chain reaction (qRT-PCR) was performed for TNF-α, IL-1β, IL-6, monocyte chemoattractant protein-1 (MCP-1), intercellular adhesion molecule-1 (ICAM-1), ionized calcium-binding adaptor molecule 1 (Iba-1), SRY-box transcription factor 2 (SOX2), endothelial nitric oxide synthase (eNOS), and neuroepithelial stem cell protein (Nestin). Glyceraldehyde-3-phosphate dehydrogenase (GAPDH) was used as the control. The collected RNA was amplified to analyze the effect of the treatment on inflammatory responses and endothelial functions.

#### Western blotting

Proteins were extracted from the isolated ischemic tissue of the left hemispheres to investigate tight junction protein expression patterns. The proteins were separated by gel electrophoresis, transferred to a membrane, and incubated with the antibodies Cav-1, claudin-5, JAM-A (Abcam, Cambridge, MA, USA), occludin (Thermo Fisher Scientific, Rockford, IL, USA), matrix metalloproteinase (MMP), and β-actin (Santa Cruz Biotechnology, Santa Cruz, CA, USA). The membrane was then stained with horseradish peroxidase-tagged secondary antibodies. Protein bands were detected using a Western blotting luminol reagent. Expression levels were quantified using ImageJ software.

#### Evans blue and fluorescence imaging

Vascular permeability was examined by monitoring the extravasation of Evans blue using a fluorescence imaging system (IVIS® Lumina S5; PerkinElmer, Hopkinton, MA, USA) to image the brain of rats. A 4% Evans blue solution was injected intravenously, and the animals were sacrificed after 2 h by intracardiac perfusion with saline to remove the circulating fluids and dye. The brain was extracted, sliced into 2-mm-thick sections, and imaged. The degree of vascular permeability was determined by quantifying pixel intensities associated with the leakage of Evans blue in the brain.

#### BrdU administration and immunofluorescence imaging

5-Bromo-2′-deoxyuridine (BrdU; Thermo Fisher Scientific, Rockford, IL, USA) was injected intraperitoneally (50 mg/kg; 10 mg/ml) to label proliferating cells for 3 d after the study. The brain was isolated, parasitized, and sectioned for further staining with other markers. Paraffin-embedded sections were deparaffinized, rehydrated in a gradient manner with ethanol, and recovered for antigens. After blocking the sections for unspecific binding, the sections were stained for cleaved caspase-3 (CC3; Thermo Fisher Scientific, Rockford, IL, USA) for the detection of neuronal apoptosis, neuronal nuclei (NeuN; ABN78; EMD Millipore, Burlington, MA, USA) for the detection of mature neurons, and doublecortin (DCX) (Thermo Fisher Scientific, Rockford, IL, USA) for the detection of neuronal progenitor cells. The sections were counterstained with secondary antibodies, and they were dehydrated in a gradient manner with ethanol, mounted, and imaged. The optical fractionator method was used to count double-labeled cells along the hippocampal regions (BrdU/NeuN-positive, BrdU/DCX-positive, or CC3/NeuN-positive cells). CC3/NeuN was stained on sections isolated on the 7th day, BrdU/DCX was stained on sections isolated on the 14th day, and BrdU/NeuN was stained on sections isolated on the 28th day.

### Statistical analysis

All statistical analyses were performed using GraphPad Prism (version 9.0.0) and SPSS (version 29.0; IBM Corporation, Armonk, NY, USA). Data distribution normality was evaluated using the Shapiro–Wilk test. For parametric data, one-way analysis of variance (ANOVA) followed by Tukey’s multiple comparison tests was used, and results are presented as mean ± standard error of the mean. ANOVA with Welch’s correction was employed for data with unequal variances, followed by Dunnett’s multiple comparisons. Nonparametric data were analyzed using the Kruskal–Wallis test followed by Dunn’s post hoc analysis, and they are presented as median [interquartile range]. The infarct volumes, rotarod test results, and neurologic deficit scores, all obtained from repeated trials, were analyzed using 2-way ANOVA for repeated measures. This analysis was conducted to determine the interaction effect between treatment and time (treatment × time) and the main effects of experimental treatment and time, followed by Tukey’s post hoc test. Greenhouse–Geisser estimates were used to correct for violations of the sphericity assumption. The sample size of each experiment was determined empirically based on findings from our prior study. *P* < 0.05 was considered statistically significant (*P < 0.05, **P < 0.01, ***P < 0.001, and ****P < 0.0001).

## Results

### Synthesis and characterization of ROS-responsive ChiBil-Statin

**Fig. 2.**
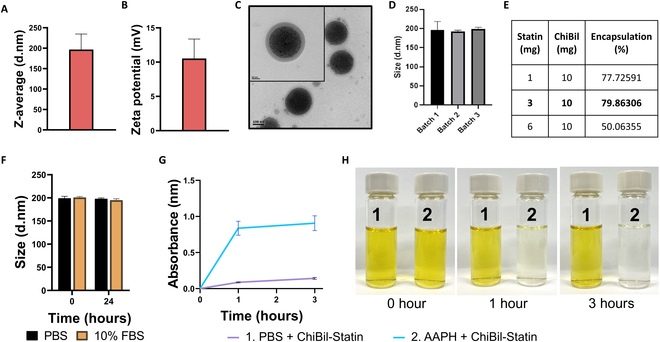
Nanoparticle characterization. (A) Size and (B) zeta potential of ChiBil-Statin nanoparticles. (C) TEM image of ChiBil nanoparticles loaded with SPIONs showing the micellar structure and size of ChiBil. (D) Three independent batches show comparable interbatch reproducibility. (E) Encapsulation efficiency of atorvastatin calcium in ChiBil evaluated by loading 1, 3, and 6 mg of statin in the same amount of micelles of 10 mg. (F) Nanoparticle stability shown in different conditions using PBS and 10% FBS for 24 h. (G) UV–visible spectra of atorvastatin calcium release with and without ROS stimuli (100 mM AAPH) in normal PBS at pH 7 and 37 °C. (H) Images showing the oxidation of ChiBil-Statin before and after incubation with a ROS generator.

The hydrodynamic size and zeta potential of ChiBil-Statin were approximately 200 nm and 9.6 mV, respectively (Fig. 2A and B). The morphology and size of the nanoparticles were confirmed with TEM (Fig. 2C), where the core was loaded with SPIONs as the negative stain. The size was ~200 nm in diameter with a 20-nm-thick micellar structure. Three independent batches showed comparable interbatch reproducibility (Fig. 2D). The critical micellar concentration (CMC) needed for polymer micelle formation was 0.46 mg/ml [10]. The encapsulation efficiency of statin in the same quantity of micelles (10 mg) was evaluated by loading 1, 3, and 6 mg of statin, which was found to be 77.72%, 79.86%, and 50.06%, respectively (Fig. 2E). When 10 mg of ChiBil micelles was loaded with 6 mg of atorvastatin, 3.036 mg was successfully encapsulated. This absolute loading amount exceeds the 2.3958 mg achieved with the same quantity of micelles exposed to 3 mg of the drug. However, a comparative analysis of encapsulation efficiency and drug wastage revealed that the 3-mg loading condition is more efficient for intravenous therapy. Next, the stability of the nanoparticle was checked in different conditions using 10% FBS and showed no change in particle size for up to 24 h (Fig. 2F). The ROS-responsive release of ChiBil showed the immediate release of statin in the presence of a ROS generator (AAPH) within 1 h (Fig. 2G) [10]. The color change in ChiBil-Statin with AAPH compared with ChiBil-Statin without AAPH showed the oxidation of bilirubin nanoparticles (Fig. 2H).

### In vitro effects of ROS-responsive ChiBil-Statin

**Fig. 3.**
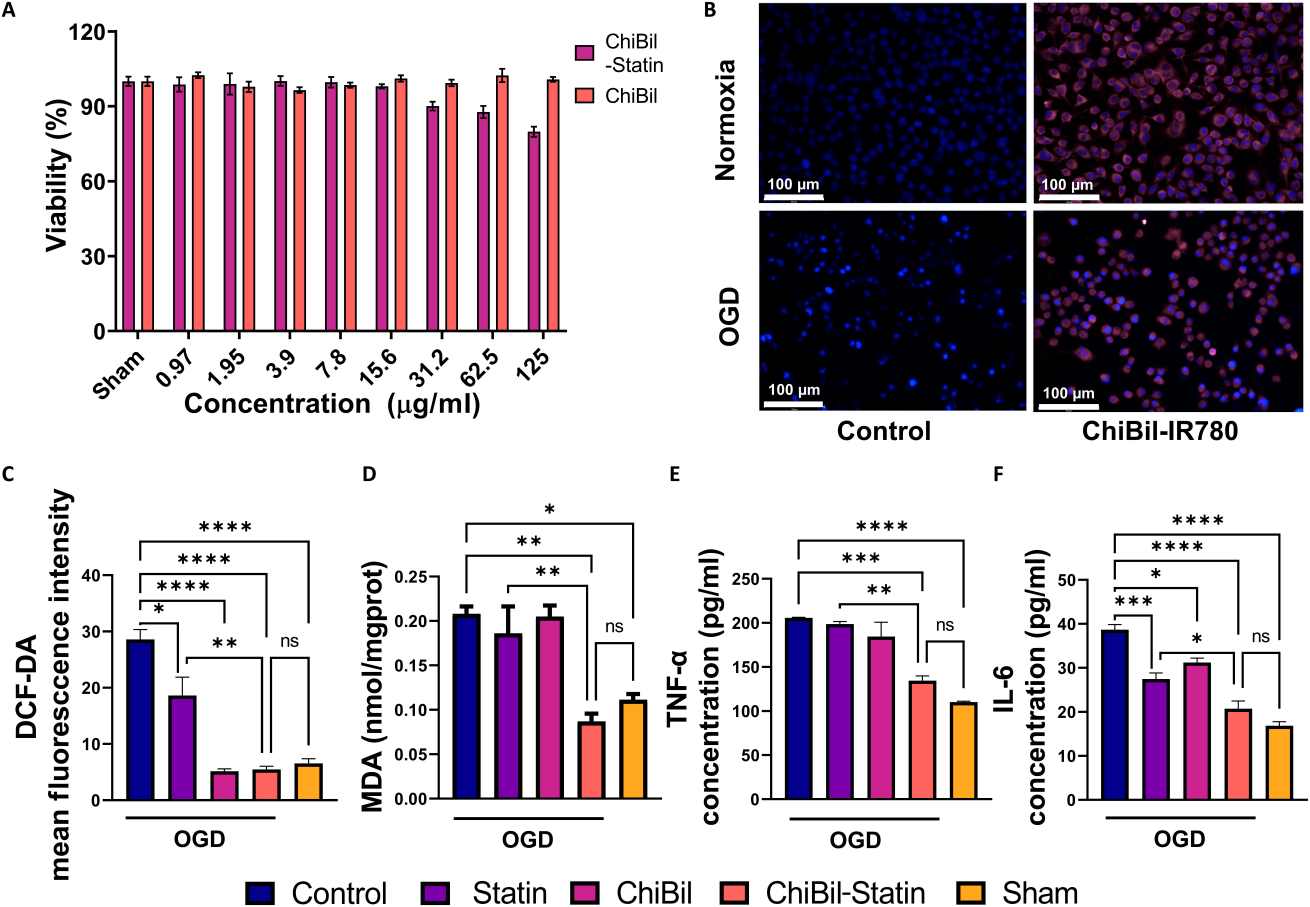
In vitro experiments for the effects of ChiBil-Statin. (A) MTS assay of the cell viability of BV-2 cells exposed to ChiBil and ChiBil-Statin for 24 h. (B) Cellular uptake study of BV-2 cells treated with ChiBil nanoparticles loaded with IR780 for 4 h under normal and OGD conditions. Scale bar, 100 μm. (C) ROS-quenching effects of ChiBil-Statin measured under OGD conditions after incubation with DCF-DA. (D) Lipid peroxidation levels were assessed by MDA detection using lysed cell samples. (E) TNF-α and (F) IL-6 levels were detected in the supernatant of BV-2 cells. Data are presented as mean ± SEM. Data were analyzed by one-way ANOVA. *n* = 3. *P < 0.05; **P < 0.01; ***P < 0.001; ****P < 0.0001.

Cell viability was assessed with different concentrations of nanoparticles loaded with and without the drug (statin) for 24 h in BV-2 cells by MTS assay (Fig. 3A). The cells showed 80% viability even at a particle concentration of 125 μg/ml. The concentration used in other studies was lower than the viable concentration of 100 μg/ml. Cell uptake studies (Fig. 3B) showed the uptake of nanoparticles under both normal and OGD conditions. ROS reduction assay (Fig. 3C) and MDA assay (Fig. 3C) were performed after treatment with PBS, statin, ChiBil, ChiBil-Statin, and sham. In comparison with OGD Control group, the ChiBil-Statin group showed around 5.6 times lower ROS production and 2.5 times lower lipid peroxidation (Fig. 3C and D) (*P* = 0.0022).

Anti-inflammatory responses were analyzed by measuring the secretion of cytokines from the cells. The OGD Control group showed elevated levels of both TNF-α (205.8 pg/ml) and IL-6 (38.67 pg/ml). The group treated with ChiBil-Statin (TNF-α = 134.5 pg/ml, IL-6 = 20.75 pg/ml) showed a reduction in the levels of inflammatory cytokines, which were similar to levels in the sham group (TNF-α = 110.1 pg/ml, IL-6 = 16.83 pg/ml), demonstrating the treatment efficacy of ChiBil-Statin. The effects of the ChiBil-Statin group were comparable to those of the Statin group (TNF-α = 198.6 pg/ml, IL-6 = 27.48 pg/ml). Taken together, the results demonstrated that the ChiBil-Statin group showed significant anti-inflammatory effects by reducing both TNF-α and IL-6 (Fig. 3E and F) (*P* = 0.0005, *P* < 0.0001).

### In vivo experiments

#### In vivo MRI

**Fig. 4.**
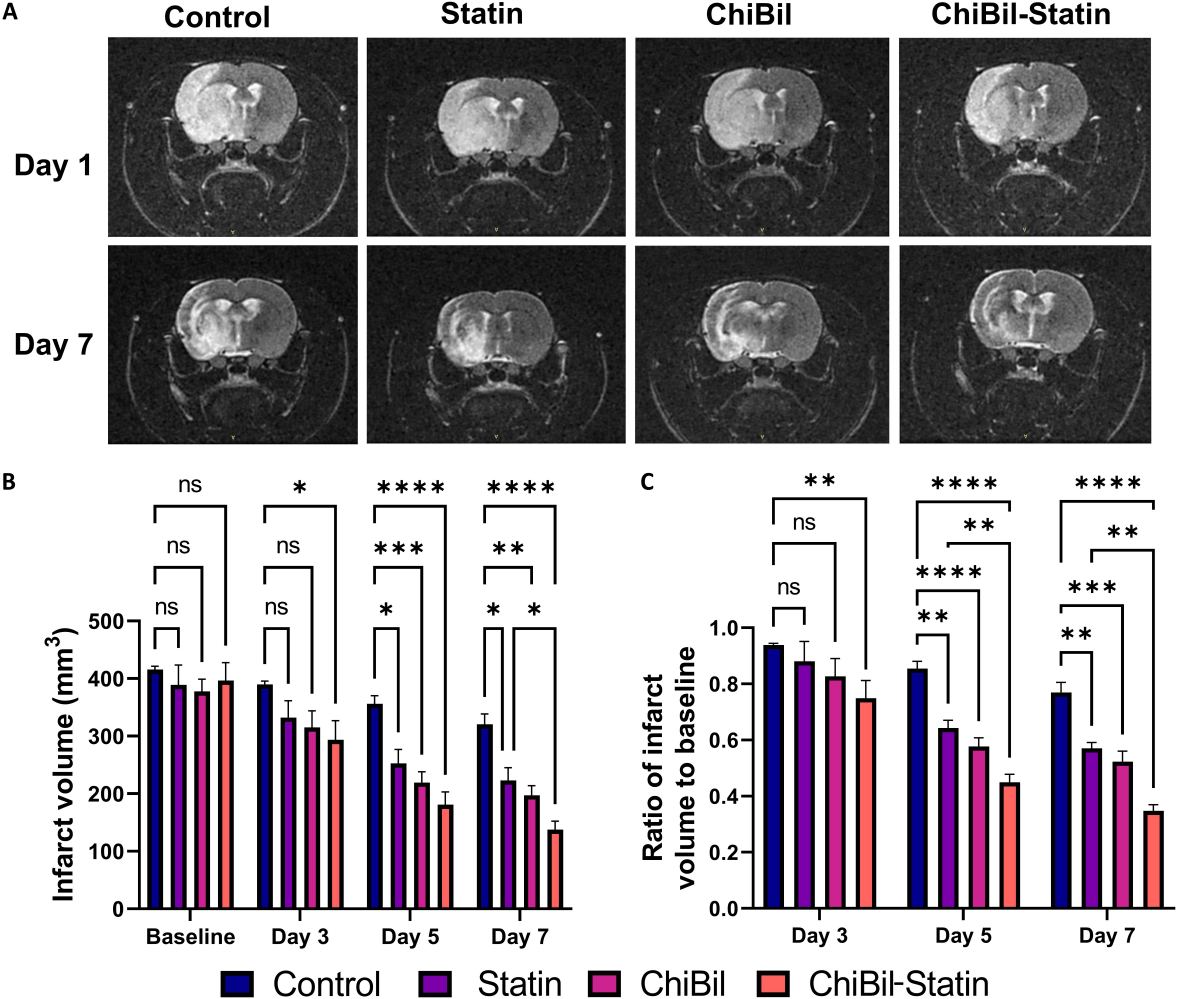
In vivo micro-MRI analysis of infarct volume. (A) MR images of ischemic rats were imaged from day 1 to day 7 on every alternate day. (B) Quantified infarct volume in ischemic regions on different days shows a significantly lower infarct volume in the ChiBil-Statin group than in the Control group (*P* = 0.013, Tukey’s post hoc analysis of repeated-measures ANOVA). Group average values of the infarct volume at 7 d after stroke (Control: 320.81 ± 50.10 mm^3^; Statin: 137.65 ± 41.13 mm^3^; ChiBil: 197.15 ± 47.49 mm^3^; ChiBil-Statin: 137.65 ± 41.13 mm^3^). (C) The ratio of infarct volume to baseline across different groups on different days shows a significant difference in the ChiBil-Statin group starting from day 3. Group average values of the ratio of infarct volumes to baseline at 7 d after stroke (Control: 0.769 ± 0.101; Statin: 0.570 ± 0.060; ChiBil: 0.523 ± 0.106; ChiBil-Statin: 0.348 ± 0.061). Data are presented as mean ± SEM. Data at each time point were analyzed using ANOVA with Tukey’s multiple comparisons. *n* = 8 in each group. *P < 0.05; **P < 0.01; ***P < 0.001; ****P < 0.0001.

Infarct volumes on T2-weighted MR images were measured every other day from day 1 to day 7. The MR images showed a significant reduction in infarction in the ChiBil-Statin group compared to that in the Control group (Fig. 4A). Infarct volumes on MR images were plotted to show the differences in volume reduction between the groups. Figure 4B shows that repeated-measures ANOVA for the infarct volume exhibited a significant interaction between treatment and time (*F*_3,30_ = 3.812, *P* = 0.020) with the main effect of treatment (*F*_3,30_ = 5.745, *P* < 0.001). The infarct volume was significantly lower in the ChiBil-Statin group than in the Control group (*P* = 0.013, Tukey’s post hoc analysis).

After 7 d, the mean infarct volume in the ChiBil-Statin group (137.65 ± 41.13 mm^3^) was significantly reduced compared to that in the Control (320.81 ± 50.10 mm^3^) and Statin (223.16 ± 61.77 mm^3^) groups (*P* < 0.01).

Day-wise comparison of infarct volumes showed no significant difference on day 3 between the Control, Statin, and ChiBil groups. However, the ChiBil-Statin group showed a significant lower infarct volume compared with the Control group (*P* = 0.0189). Although a reduction in infarction was observed in all groups on days 5 and 7, the reduction in the ChiBil-Statin group was the most statistically significant compared with that in the Control group (Fig. 4B and C) (*P* < 0.0001). As shown in Fig. 4C, the ratio of the infarct volume at 7 d was significantly lower in the ChiBil-Statin group (*P* < 0.0001) than in the Control group. In the Statin group (*P* = 0.0046) and ChiBil group (*P* = 0.0003), the ratio of the infarct volume at 7 d was also lower than in the Control group. The day-wise comparison of the ratio of infarct volume to baseline showed a significant difference on day 3 only in the ChiBil-Statin group compared with the Control group. Similar to the results for infarct volume, the ratio in the ChiBil-Statin group was significantly lower than in the Statin group on days 5 and 7 (*P* < 0.008).

#### Behavioral study

**Fig. 5.**
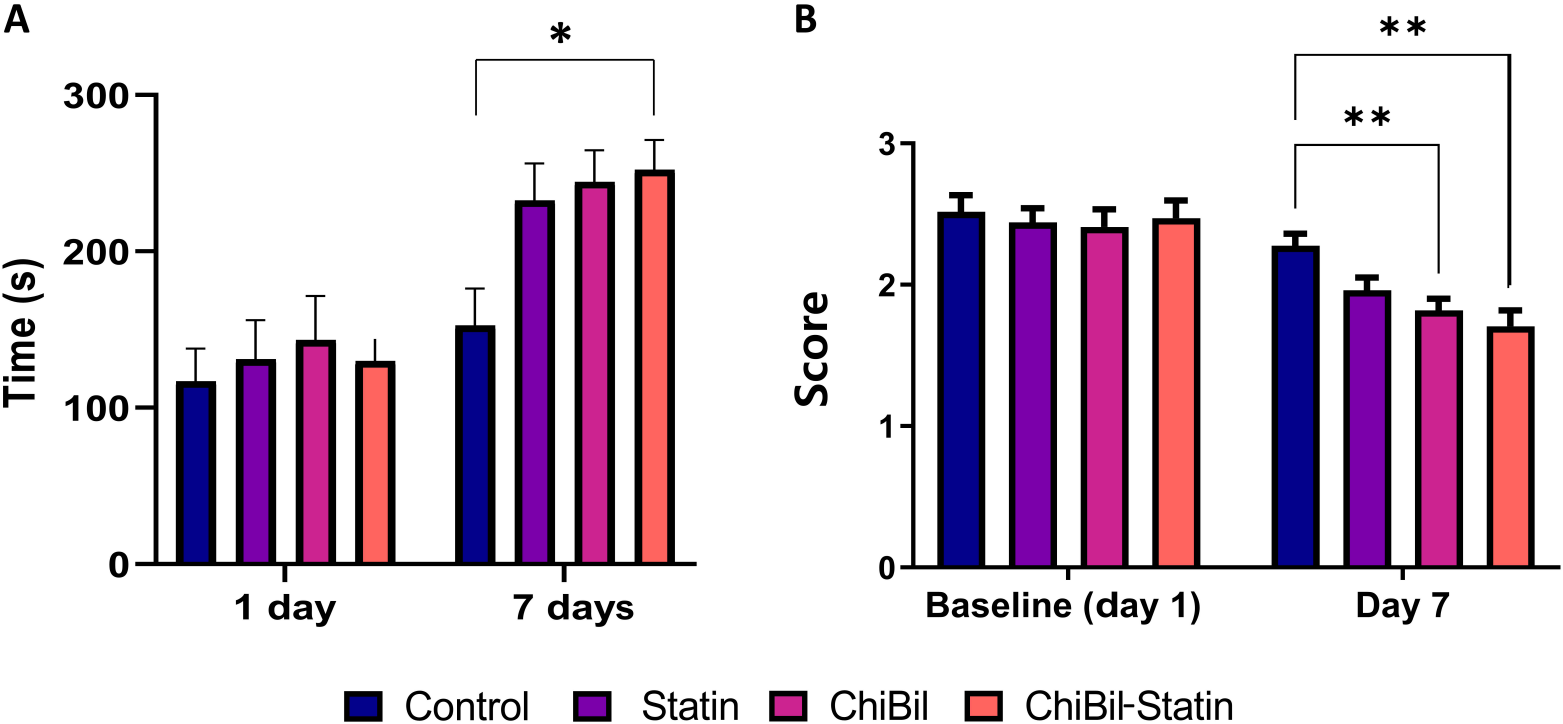
Behavioral assessment of cerebral ischemia-induced rats according to the treatment. (A) Rotarod test results show a significant interaction between treatment and time based on their coordination, balance, and endurance (*P* = 0.024; repeated-measures ANOVA). The ChiBil-Statin group demonstrated significantly longer times of fall than the Control group (*P* = 0.042, post hoc analysis using Tukey’s test). (B) Neurologic deficit scores for the different groups on days 1 and 7. Repeated-measures ANOVA for the NDS shows lesser neurological deficit for the ChiBil-Statin (*P* = 0.002) and ChiBil (*P* = 0.006) groups than for the Control group (Tukey’s post hoc analysis). Data are presented as mean ± SEM, with *n* = 17 in each group. The number of animals that died during the 7-d treatment was 12, 8, 5, and 3 in the Control, Statin, ChiBil, and ChiBil-Statin groups, respectively. *P < 0.05; **P < 0.01.

The rotarod test was used to assess the coordination, balance, and endurance of rats. Posttreatment times to fall in the ChiBil-Statin group were significantly longer than those in the Control group, suggesting enhanced motor function (treatment × time interaction effect, *F*_3,64_ = 3.358, *P* = 0.024; main effect of time, *F*_1,64_ = 32.616; *P* < 0.001; main effect of treatment, *F*_3,64_ = 2.042, *P* = 0.117; 2-way ANOVA for repeated measures). Specifically, the ChiBil-Statin group showed a significant improvement compared to the Control group (*P* = 0.042, Tukey’s post hoc analysis; Fig. 5A).

In NDS, ChiBil-Statin and ChiBil treatments for 7 d led to a significantly lower neurological deficit (treatment × time interaction effect, *F*_3,64_ = 5.858, *P* = 0.001; 2-way ANOVA for repeated measures). The behavior test suggests enhanced neurological recovery for the ChiBil-Statin and ChiBil groups (*P* = 0.002 and *P* = 0.006 compared to the Control group, respectively, Tukey’s post hoc analysis; Fig. 5B).

#### qRT-PCR for inflammatory, endothelial, and neurogenesis markers

**Fig. 6.**
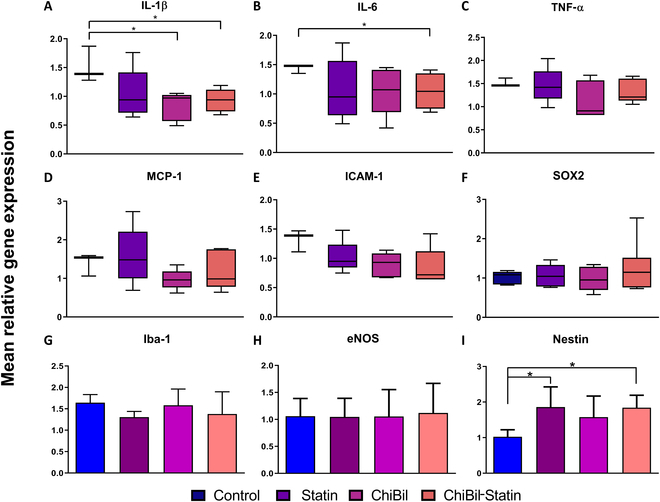
RT-PCR for inflammatory, endothelial, and neurogenesis markers. (A) IL-1β, (B) IL-6, (C) TNF-α, (D) MCP-1, (E) ICAM-1, (F) SOX2, (G) Iba-1, (H) eNOS, and (I) Nestin mRNA expression. The ChiBil-Statin treatment led to decreased gene expression of IL-1β, IL-6, and ICAM-1, along with increased gene expression of Nestin compared to that in the Control group. The ChiBil treatment led to decreased gene expression of IL-6 compared to the Control group. The statin treatment led to increased gene expression of Nestin compared to that in the Control group. Nonparametric data are analyzed using the Kruskal–Wallis test with Dunn’s post hoc analysis and are presented as median with interquartile range, with minimum to maximum whiskers in box plots. Parametric data are analyzed using one-way ANOVA with Tukey’s post hoc analysis and presented as mean ± SEM. *n* = 6 in each group. *P < 0.05; **P < 0.01.

The expression levels of pro-inflammatory factors and adhesion molecules were evaluated to quantify gene expression levels after ChiBil-Statin therapy (Fig. 6). The expression of inflammatory cytokines and chemokines such as IL-1β and IL-6 showed a significant reduction in the ChiBil-Statin group compared with the Control group. IL-1β levels were also significantly lower in the ChiBil group than in the Control group. ICAM-1, a cell adhesion molecule responsible for neurological deterioration, showed higher gene expression in the Control group than in the ChiBil-Statin group. Additionally, Nestin, a marker for central nervous system (CNS) progenitor cells, showed a significant increase in expression in the ChiBil-Statin and Statin groups compared to that in the Control group.

#### Western blotting for tight junction proteins and IVIS® imaging for BBB integrity

The treatment of cerebral ischemia with ChiBil-Statin led to the elevated expression of tight junction proteins, indicating restored BBB function. In comparison with the Control, Statin, and ChiBil groups, the ChiBil-Statin group showed the elevated expression of occludin, which is involved in maintaining BBB integrity and reducing BBB permeability. On the other hand, Cav-1 prevents tight junction protein degradation by inhibiting MMPs, thus protecting BBB integrity [28]. The results showed the significantly increased level of Cav-1 in the ChiBil-Statin group compared with the Control, Statin, and ChiBil groups after treatment.

**Fig. 7.**
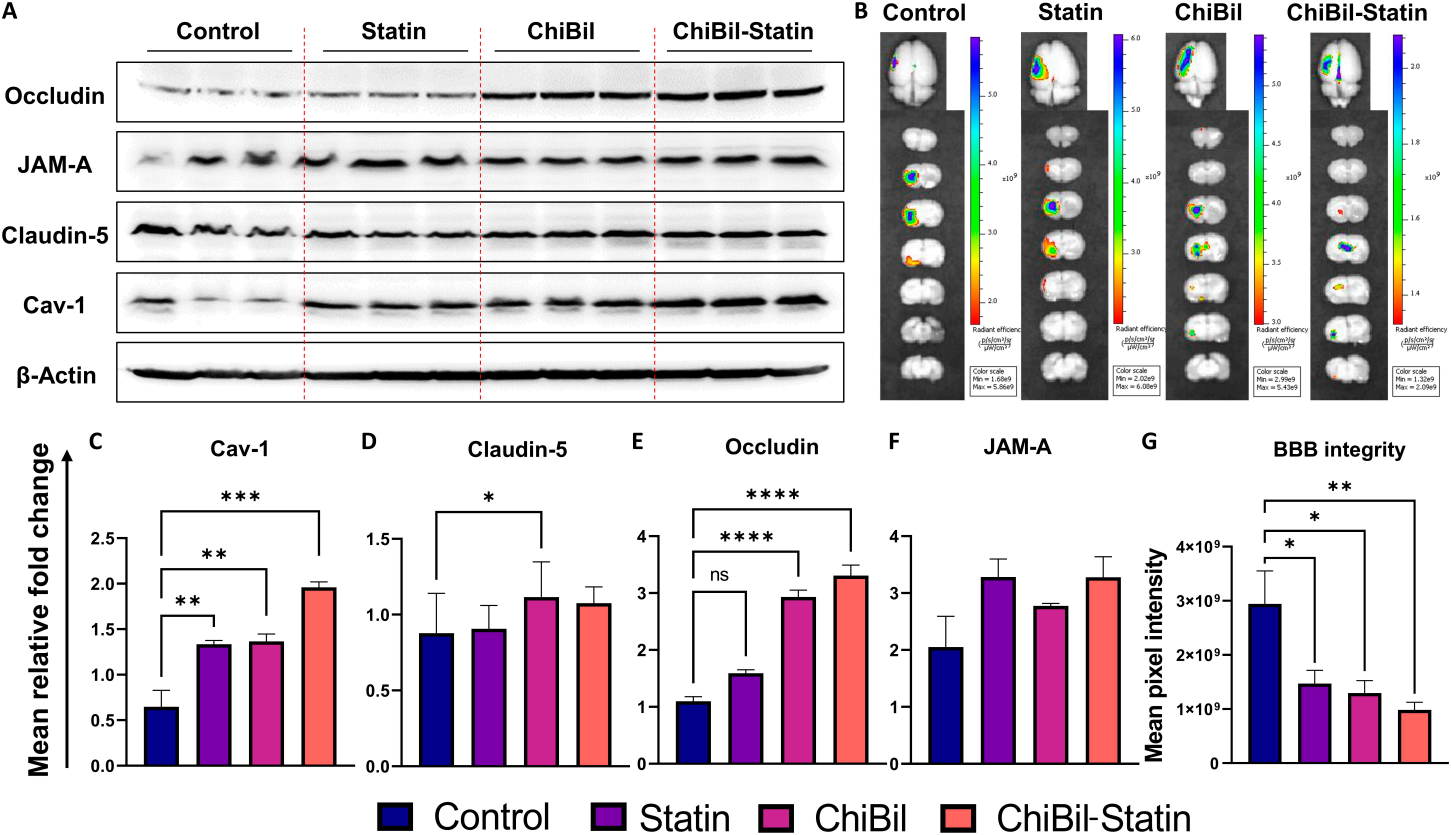
Western blotting and IVIS® imaging for BBB integrity. (A) Images of Western blot. (B) IVIS® fluorescence images of ischemic rat brain on day 7. Western blot quantification of (C) Cav-1, (D) claudin-5, (E) occludin, and (F) JAM-A. The ChiBil-Statin treatment increased tight junction and regulatory protein expression of Cav-1, claudin-5, occludin, and JAM-A compared to the Control group. The ChiBil treatment also led to higher protein expression of claudin-5 and occludin than those in the Control group. The statin treatment led to increased protein expression of JAM-A compared to that in the Control group. (G) Quantified data of fluorescence accumulation reflecting the extent of BBB disruption in the ischemic parts. The ChiBil-Statin, ChiBil, and Statin treatments show decreased BBB disruption compared to the Control group, with the lowest disruption observed in the ChiBil-Statin group. Parametric data are analyzed using one-way ANOVA with Tukey’s post hoc analysis and presented as mean ± SEM. *n* = 6 in each group. *P < 0.05; **P < 0.01; ***P < 0.001; ****P < 0.0001.

A comparison of the ischemic regions of all 4 groups showed that the Control group had the highest pixel intensity, followed by the Statin group, ChiBil, and ChiBil-Statin groups in IVIS® imaging (Fig. 7F). The mean pixel intensity, which reflects the extent of BBB disruption, was significantly higher in the Control group (2.94 × 10^9^ p/s/cm^2^) than in the Statin (1.47 × 10^9^ p/s/cm^2^), ChiBil (1.29 × 10^9^ p/s/cm^2^), or ChiBil-Statin (0.98 × 10^9^ p/s/cm^2^) groups. However, there was no significant difference among the Statin, ChiBil, and ChiBil-Statin groups.

#### Immunofluorescence imaging for apoptosis and neurogenesis

**Fig. 8.**
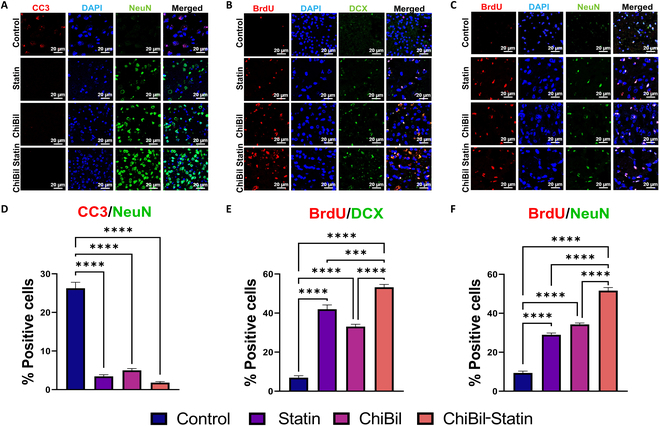
Immunofluorescence staining on ischemic penumbra in the subventricular zone. Brain tissues stained for (A) CC3 (red)/NeuN (green), (B) BrdU (red)/DCX (green), and (C) BrdU (red)/NeuN (green). Scale bar, 20 μm. (D) A CC3/NeuN-positive population in the ischemic region shows a quantitative percentage of apoptotic neurons on day 7. The number of CC3/NeuN-positive cells is significantly lower in the ChiBil-Statin group than in the Control, Statin, and ChiBil-Statin groups. (E) A quantitative percentage of survival of newly generated cells was shown by BrdU/DCX-positive cells in the ischemic regions on day 14. BrdU/DCX-positive cells are significantly higher in the ChiBil-Statin group than in the Control, Statin, and ChiBil groups. (F) BrdU/NeuN-positive cells in the ischemic regions showed a quantified percentage of neurogenesis on day 28. BrdU/NeuN staining shows markedly improved survival of newly generated neurons in the ChiBil-Statin group compared to that in the Control, Statin, and ChiBil groups. Parametric data are analyzed using one-way ANOVA with Tukey’s post hoc analysis and presented as mean ± SEM. *n* = 6 in each group. *P < 0.05; **P < 0.01; ***P < 0.001; ****P < 0.0001.

Compared with the saline treatment, ChiBil-Statin treatment exhibited better neuroprotective effects (Fig. 8). CC3, BrdU, DCX, and NeuN staining in ischemic regions demonstrated the neuroprotective effects of ChiBil-Statin. CC3 is a marker of apoptosis; thus, a combination of CC3/NeuN can facilitate the identification of apoptotic neurons. The staining results for CC3/NeuN showed that the percentage of apoptotic neurons was significantly lower in the ChiBil-Statin group (1.805%) than in the Control group (26.26%). Furthermore, the apoptotic neuron percentage in the ChiBil-Statin group was significantly lower than in the Statin (3.433%) and ChiBil (4.985%) groups. BrdU/DCX staining indicated that the percentage of newly generated cells in the subventricular zone on day 14 of treatment was significantly higher in the ChiBil-Statin group (53.22%) than in the Control group (6.908%). BrdU/DCX results also indicated that the percentage of the newly generated cells was significantly higher in the Statin (41.99%) and ChiBil (33.07%) groups than in the Control group. BrdU/NeuN staining was performed to examine the survival of generated neurons on day 28, revealing markedly improved survival rates of these neurons in the ChiBil-Statin group compared to those in the Control, Statin, and ChiBil groups in the subventricular zone. However, no significant difference was observed between the Statin and ChiBil groups.

## Discussion

Our study demonstrated that ROS-responsive ChiBil-Statin could be effectively delivered to the ischemic brain, exerting marked synergistic pleiotropic neuroprotective effects. In vitro experiments showed the uptake of ChiBil-Statin and a reduction in intracellular ROS, lipid peroxidation, and inflammatory cytokines. In vivo experiments showed that ChiBil-Statin improved motor function recovery and decreased infarct volumes in cerebral ischemia–reperfusion injury. ChiBil-Statin also reduced inflammatory cytokines and apoptosis in the penumbra area. In addition, ChiBil-Statin enhanced endothelial functions, BBB integrity, and neurogenesis in the subventricular zone. Our results suggest that ChiBil-Statin holds promise as a targeted therapy for ischemic stroke characterized by increased ROS production. Given the importance of neuroprotection as the most essential residual unmet need among patients with ischemic stroke, the current results may open up new avenues for future research and potential clinical applications.

The potential of ROS-responsive nanoparticles in reducing oxidative stress, neuronal apoptosis, brain edema, and infarct volume has been established in animal models and preclinical studies related to cerebral ischemia [29,30]. ROS-responsive nanoparticles hold promise as a novel and targeted approach for mitigating oxidative damage and improving outcomes in ischemic stroke patients [30–32]. The mechanisms underlying the improvement of ischemic conditions following treatment with ROS-responsive nanoparticles might involve the reduction of ROS, which in turn reduces inflammation in the ischemic regions, leading to infarct volume reduction and neurogenesis [30–33]. Additionally, these nanoparticle formulations can enhance the stability, bioavailability, and BBB permeability of antioxidant agents, overcoming the limitations of conventional therapies [30,31,34]. Atorvastatin has also been reported to reduce infarct volume due to inhibitory effects on inflammation and oxidative stress, facilitating the recovery of BBB breakdown and endothelial dysfunction [18–24].

Our research, in line with previous studies, underscores the unique potential of ROS-responsive ChiBil-Statin nanoparticles. These nanoparticles, loaded with atorvastatin, have shown a remarkable ability to reduce cerebral infarct volume and neurological deficit while also demonstrating anti-inflammatory properties. Their size, approximately ~200 nm, makes them ideal for targeted delivery to ischemic regions, thereby enhancing their therapeutic effects in treating cerebral ischemic injuries characterized by increased ROS production [35]. Notably, our study revealed their effective drug release in the presence of the ROS generator AAPH. We chose to load atorvastatin, a hydrophobic statin known for its ability to cross the BBB and exert neuroprotective effects [23,24]. Atorvastatin treatment resulted in reduced infarct volume, increased expression of CNS progenitor cells and neurogenesis, as well as decreased BBB breakdown and apoptosis. However, statin treatment alone showed limited effect on ROS reduction, behavioral recovery, anti-inflammation responses, and increased tight junction protein expression.

Our findings indicate that ChiBil nanoparticles can enhance the limited efficacy of statin. ChiBil showed ROS-reducing capability, reduced lipid peroxidation and inflammatory markers, improved neurological deficit, and increased tight junction protein expression. Therefore, the beneficial effects observed in the ChiBil-Statin group may be attributed to the combined effects of statin and ChiBil, given that the ChiBil group showed minimal differences compared with the Control group. Owing to the synergistic effect of ROS-responsive ChiBil nanoparticles and atorvastatin, ChiBil-Statin could reduce the volume of cerebral infarction from day 3 of the treatment regimen even though the concentration of statin was twice or 3 times lower than that previously used [18–24]. Moreover, the bioavailability and the general circulation of statin drugs are relatively low after oral administration due to clearance by the digestive system, poor solubility, and low permeability [36]. When administered intravenously, the same effect can be achieved with a lower dose than when taken orally [37]. Nanoparticle drug delivery systems can improve bioavailability and therapeutic efficacy [36]. Our findings suggest that intravenous ROS-responsive ChiBil-Statin therapy may serve as a promising synergistic therapy for neuroprotection, effectively targeting the affected area while minimizing off-target release.

The BBB can become compromised during cerebral ischemia, leading to increased permeability. Several factors contribute to BBB disruption, such as endothelial cell damage, tight junction disruption, inflammatory responses, and elevated MMPs or ROS [38]. Compromised BBB permeability during cerebral ischemia has important clinical implications. It allows the entry of inflammatory cells, proteins, and other molecules into the brain, which can exacerbate tissue damage and contribute to secondary injury [39]. Additionally, increased BBB permeability may reduce the efficacy of pharmacological treatments by affecting drug delivery to the brain. In our study, compared with saline and statin treatment, ChiBil-Statin treatment improved the integrity of the BBB, as demonstrated by the results of Western blotting and IVIS® imaging.

During ischemic stroke, the concentration of ROS increases from normal low levels to a peak level during reperfusion, possibly underlying apoptosis [40]. The expression of the apoptotic markers CC3/NeuN was significantly lower in all 3 groups (statin, ChiBil, and ChiBil-Statin) compared with the Control group on day 7. These results indicated that ChiBil-Statin, statin, and ChiBil may exert neuroprotective effects by reducing apoptosis. However, neurogenesis was significantly higher in the ChiBil-Statin group than in all other groups. BrdU is a marker of cell proliferation, which binds to the DNA of dividing cells in the S phase, and DCX is often used as a gold standard marker of newly developed cells [41]. The immunofluorescence of BrdU/DCX and BrdU/NeuN showed a significant increase in neurogenesis in all 3 groups (statin, ChiBil, and ChiBil-Statin) compared with the Control group. Notably, the comparison between ChiBil-Statin and statin or ChiBil alone demonstrated the neurogenesis effects of ChiBil-Statin. ChiBil or statin alone may have neuroprotective effects; however, the results showed greater improvements with ChiBil-Statin. Overall, the findings indicated the synergistic effects of ChiBil-Statin on cerebral ischemia.

Our study indicates that ChiBil nanoparticles can be a multifunctional, ROS-sensitive nanocarrier to improve drug delivery efficacy and enhance neuroprotection. Drug delivery to the brain is constrained owing to protective barriers, specifically the BBB, which restricts the passage of foreign particles into the brain. Nanoparticles, such as hydrogels, exosomes, quantum dots, and liposomes, can cross the BBB and deliver drugs to lesion sites [42]. Liposome nanocarriers have long been employed in drug delivery, demonstrating efficiency in delivering therapeutic molecules in ischemic stroke therapy [43]. However, no currently marketed liposomal curative agents have shown a significant overall survival benefit when directly compared to traditional stroke drugs, which may be attributed to factors such as lipid instability, drug leakage, and insufficient targeting of lesion sites [44]. Micellar nanoparticles exhibit therapeutic potential in drug delivery and are considered a potential liposome alternative [30]. Our ROS-responsive ChiBil nanoparticles offer scalability, especially for neurological disease, as their hydrophobic core can be loaded with hydrophobic drugs that effectively cross the BBB, such as atorvastatin or other emerging neuroprotective agents [45,46]. Additionally, the hydrophilic shell renders the whole system water soluble, allowing rapid and uniform distribution throughout the body. This design stabilizes the core, enhances blood circulation time, and provides ROS-sensitive ability, ultimately resulting in targeted delivery and improving therapeutic efficacy [46].

Nanomedicine has revolutionized the discovery and administration of drugs in biological systems [47]. Although nanotechnology is not approved for treating stroke, numerous preclinical studies and limited clinical trials offer hope and opportunities to translate these innovations into practical clinical tools [48]. The number of US Food and Drug Administration-approved nanotechnology-based products and clinical trials has increased recently. The leading categories include liposomes, lipid-based nanoparticles, polymers, and nanocrystals, often used in combination with drugs or biologics, primarily in cancer treatment [49]. However, future research may increasingly focus on smart nanoparticles for precise targeting of the lesion area in the field of neurological disease [30,31]. Our smart targetable ChiBil-Statin nanoparticles can be optimized for large-scale production based on the CMC under good manufacturing practice conditions for clinical application [50]. Our findings suggest that ROS-responsive multifunctional ChiBil-Statin nanoparticles may be a promising candidate for a novel therapeutic strategy for stroke as a pleiotropic neuroprotective agent and have the potential for application in clinical trials and real-world practice.

This study has several limitations. First, we could not examine the biodistribution, ROS responsiveness, or the amount of statin present in the brain after crossing the BBB in vivo. Nevertheless, we demonstrated cell uptake, ROS-quenching effects, and lipid peroxidation and inflammatory marker reduction in vitro following ChiBil-Statin treatment. Extended research on the biodistribution of ChiBil-Statin is warranted. Second, the anatomy, physiology, and genetic differences between species might limit the translation of the results from bench to bedside. However, it is expected that ROS-responsive ChiBil-Statin may be used as a promising therapeutic for patients with acute ischemic stroke and other ischemic vascular diseases characterized by increased ROS production. Additional studies with humans are needed to determine the safety of the nanoparticles before they can be used in clinical settings.

In conclusion, we demonstrated the beneficial effects of ROS-responsive ChiBil-Statin on ischemia–reperfusion injury. Our results provide compelling evidence showing the synergistic pleiotropic neuroprotective effects of multifunctional ChiBil-Statin to reduce brain injury and promote recovery by combining ChiBil and atorvastatin for targeted delivery to ischemic brain lesions as ROS-responsive nanoparticles. Therefore, ChiBil-Statin holds promise as a targeted therapy for ischemic stroke and other major ischemic vascular diseases characterized by increased ROS production, leading to new avenues for future research and potential clinical applications.

## Data Availability

The data supporting the current study’s findings are available from the corresponding author upon reasonable request.
